# Sleep and Professional Burnout in Nurses, Nursing Technicians, and Nursing Assistants During the COVID-19 Pandemic

**DOI:** 10.1097/jnr.0000000000000501

**Published:** 2022-06-08

**Authors:** Mariana Alvina DOS SANTOS, Flávia Helena PEREIRA, Juliano DE SOUZA CALIARI, Henrique Ceretta OLIVEIRA, Maria Filomena CEOLIM, Carla Renata Silva ANDRECHUK

**Affiliations:** 1 PhD, RN, Adjunct Professor, Federal University of Mato Grosso do Sul, Três Lagoas, Mato Grosso do Sul, Brazil; 2 PhD, RN, Professor, Federal Institute of Science, Education and Technology of the South of Minas Gerais, Passos, Brazil; 3 MSc, Statistician, Faculty of Nursing, Campinas State University, Campinas, São Paulo, Brazil; 4 PhD, RN, Associate Professor, Faculty of Nursing, Campinas State University, Campinas, São Paulo, Brazil; 5 PhD, RN, Research Collaborator, Faculty of Nursing, Campinas State University, Campinas, São Paulo, Brazil.

**Keywords:** nursing, sleep, burnout, professional, job satisfaction, emotional exhaustion

## Abstract

**Background:**

The COVID-19 pandemic may trigger sleep disorders and burnout in nursing professionals.

**Purpose:**

This study was designed to describe the occurrence of sleep disorders and burnout in a nursing team during the COVID-19 pandemic and to identify the associated factors.

**Methods:**

A cross-sectional approach was used. The questionnaire was administered via the Internet. All of the participants were nursing professionals who had provided care during the COVID-19 pandemic, and data were collected between June and August 2020. Sociodemographic and work characterization instruments, the Jenkins Sleep Scale, and the Maslach Burnout Inventory were used.

**Results:**

Five hundred seventy-two nursing professionals (nurses, nursing technicians, and nursing assistants) responded. Slightly over one quarter (26.4%) presented a sleep disorder, and 17.3% presented burnout. Professional category was a factor found to be associated with having a sleep disorder. Moreover, a lower prevalence both of disorders and of starting to use sleep medication was found among nurses than nursing assistants. Moreover, an association was found between having a high level of emotional exhaustion burnout and being a nursing technician, having a higher number of patients needing care, and starting to use sleep medication. The level of burnout related to depersonalization was significantly higher for nursing assistants, those with a weekly workload of 50 hours or more, and those starting to use sleep medication. Furthermore, burnout related to personal accomplishment was significantly higher in those starting to use sleep medication. Among the participants with sleep disorders, according to Jenkins Sleep Scale results, all of the participants presented a high or moderate level of emotional exhaustion and a high level of burnout related to personal accomplishment.

**Conclusions/Implications for Practice:**

The findings indicate that the incidence of sleep disorders and burnout were high among nursing professionals during the COVID-19 pandemic and mainly related with starting to use sleep medication. The results demonstrate the importance of detecting and assessing the frequency of sleep disorders and professional exhaustion. Interventions that aim to improve sleep quality and working conditions for these professionals should be developed.

## Introduction

Since the World Health Organization declared the disease caused by the new coronavirus COVID-19 a global pandemic, the efforts of the international scientific community have significantly increased the evidence related to this disease and its impact on the community. Preventive measures such as social distancing and quarantine were implemented, and workers were instructed to work from the home as much as possible. Nursing professionals, due to performing an essential activity, continued their work activities in their usual practice scenarios (Forte & Pires, 2020).

Nursing is an important, round-the-clock workforce on the frontlines of COVID-19 care. Brazil defines three categories of nursing professionals (nurse, nursing technician and nursing assistant), which reflect degree and length of training and work responsibilities. Despite the important role that they play, nursing professionals, especially nursing assistants and technicians, face precarious work conditions with multiple job contracts, excessive hours, and low wages (Silva et al., 2020).

The pandemic has been shown to impact the mental and physical health of individuals, with sleep being one of the most compromised aspects, especially in health professionals. In recent years, even predating the pandemic, the incidence of sleep problems has been rising among nursing professionals. The most frequent complaints among nurses have been reported as insufficient sleep (70.1%), short sleep duration (48.6%), and insomnia (36.0%; Silva-Costa et al., 2015). Studies carried out during the pandemic have reaffirmed the impact of sleep problems and poor sleep quality among nursing professionals (Khatony et al., 2020; Zeng et al., 2020; Zhou et al., 2020). A study carried out in China with 7,236 volunteers found elevated reports of anxiety disorders, depressive symptoms, and poor sleep quality during this period, with health professionals complaining more about poor sleep quality (Huang & Zhao, 2020).

External, environmental and social factors may also act as predisposing factors for poor sleep. During periods of stressful events and changes in routine, sleep quality may be negatively affected (Palagini et al., 2013). Individuals that report not having restful sleep have less control over events that may be stressful (Wiernik et al., 2014), with related impacts on their subjective well-being and satisfaction with life. Furthermore, in facing the current pandemic, professionals, especially nursing professionals, linked to the treatment of patients infected with COVID-19 have faced elevated stressors in their practice associated with shortages of personal protective equipment, lack of communication and support, isolation, social stigma, fear of transmitting illness to a loved one, and high levels of workplace stress. This has resulted in greater risks of anxiety, depression, burnout, dependency, and posttraumatic stress in health professionals (El-Hage et al., 2020).

The state of emotional tension and stress caused by exhausting work conditions may lead to professional exhaustion (aka burnout) syndrome, which is an emotional disorder with symptoms including extreme fatigue, stress, and physical exhaustion resulting from very tiring work situations that demand a high level of competitiveness or responsibility. It may lead to a profound depressive state that has quantitative and qualitative impacts on the care provided by nursing professionals (Campos & Maroco, 2012). In Italy, high levels of burnout have been observed in health professionals during the pandemic, including, in particular, emotional exhaustion and reduced personal accomplishment (Giusti et al., 2020). Nurses that worked in COVID-19 wards reported higher levels of stress, exhaustion, and depressed mood, as well as lower levels of work-related achievement compared with nurses on other wards, demonstrating a higher risk of psychological overload (Zerbini et al., 2020).

Nursing professionals face a panorama of challenges in the COVID-19 pandemic, as they undergo changes in behavior and, especially among those on the frontlines of care, must deal with the fear of becoming infected, which may negatively impact mental health and increase the risk of sleep disorders, poor sleep quality, and burnout. Therefore, personal health as well as the quality of care provided may be compromised. More information about the factors associated with sleep disorders and burnout should contribute to the development of strategies to minimize their occurrence and impact.

In light of the above, this article was developed to describe the occurrence of sleep disorders and burnout among nursing staff during the COVID-19 pandemic and to identify the related factors.

## Methods

### Participants

This was a cross-sectional, quantitative study. The selection of participants was non-probabilistic and was carried out using an online questionnaire in the northern, northeastern, southeastern, central west, and southern regions of Brazil. The inclusion criteria adopted were: 18 years old or over and employed as a nurse, nursing technician, or nursing assistant working in care provision during the COVID-19 pandemic. Otherwise, qualified individuals who worked at night only were excluded.

The estimated target sample size was 664 participants. For this calculation, a 99% confidence interval, a 5% sampling error, and an 80% power were applied, considering a heterogeneous population, using data from the Federal Nursing Council, published on April 4, 2020, in which the number of nursing professionals in Brazil was 2,304,509 (Federal Council of Nursing, 2020). The sample effectively studied (*N* = 572) was 86.1% of the targeted sample size.

Participant recruitment took place through dissemination in virtual groups, associations, and communities on various social media platforms/social networks as well as through groups of application contacts distributed throughout the national territory between June and August 2020. The link to the study with an invitation to participate was sent and those that agreed signed the consent form in digital format and were enrolled as participants in this study. The participants were then given access to the questionnaire. The online questionnaire was disseminated using the snowball technique, in which each participant was invited to further disseminate the study after their participation.

### Measures

The following sociodemographic and work data were collected: gender (male, female), age, professional training (high school, undergraduate, postgraduate/specialization, postgraduate/master's, postgraduate/doctorate, post-doc), professional category (nurse, nursing technician, nursing assistant), number of jobs (one, two or more), area of activity (primary and secondary care, tertiary and prehospital care), weekly workload (20–30 hours, 31–40 hours, 41–50 hours, more than 50 hours), and state of residence (North, Northeast, Southeast, Central West, South).

The variables of interest that were used to indicate changes during the pandemic were obtained using the following questions: “Did you see an increase in the number of patients and care provided?”, “Did you see an increase in tension and stress among the team members on duty?”, and “Did you start using sleep medications?”. The answer options of “yes” and “no” were provided for all questions.

In relation to sleep, the self-assessment of sleep quality was verified using the question “How do you rate your sleep during the pandemic?”, with three answer options including excellent, good, and bad/very bad.

The frequency and intensity of certain sleep disorders were assessed using the Portuguese version (Reis et al., 2014) of the Jenkins Sleep Scale (JSS; Jenkins et al., 1988). The instrument consists of four self-administered questions that address the frequency of sleep problems encountered during the previous month: (a) I had difficulty falling asleep, (b) I woke up several times during the night, (c) I had trouble staying asleep (including waking up very early), and (d) I woke up and felt tired or worn out even after a complete period of sleep. The response alternatives were arranged on a six-point Likert-type scale, with 0 = *never*, 1 = *a few days* (1 to 3), 2 = *some days* (4 to 7), 3 = *half the days* (8 to 14), 4 = *most days* (15 to 21), and 5 = *every day* (21 to 31). The overall score range was zero to 20 points, with a mean score of ≥ 4 on at least 15 nights during the month indicating the presence of sleep disorder. The instrument showed good reliability in this study (Cronbach's alpha = .87).

The measurement of the physical and emotional exhaustion in the participants was carried out using the Portuguese version (Pereira, 2017) of the Maslach Burnout Inventory (MBI), which was designed to assess the feelings of professionals toward their work (Maslach et al., 2001). The MBI contains 22 items distributed in three subscales: emotional exhaustion (EE: 9 items), depersonalization (DE: 5 items), and personal accomplishment (PA: 8 items). Responses are measured using a five-point Likert-type scale: 1 = *never*, 2 = *rarely*, 3 = *sometimes*, 4 = *often*, and 5 = *always*. For the EE subscale, burnout level was considered low for scores of ≤ 16, moderate for scores of 17–26, and high for scores of ≥ 27. For the DE subscale, scores of ≤ 6 were considered low, 7–12 considered moderate, and ≥ 13 considered high. The PA subscale was reverse scored, with values of ≤ 31 corresponding to a high level, 38–32 corresponding to a moderate level, and ≥ 39 corresponding to a low level of burnout. Burnout syndrome is characterized as low if the scores are low in the EE and DE and high in the PA subscales, and as high when the scores are high in the EE and DE and low in the PA subscales. In this study, the reliability values, calculated for the subscales using Cronbach's alpha, were EE = .90, DE = .63, and PA = .79.

### Ethical Considerations

This study was approved by the research ethics committee under Authorization No. CAAE 31681020.9.0000.8158 issued on May 29, 2020, and was developed following the ethical precepts of Resolution No. 466/2012 of the National Health Council.

### Statistical Analysis

The data were exported from Google Forms directly into a Microsoft Excel spreadsheet and double checked for correctness. After eliminating incomplete questionnaires, the data were transferred to SAS software Version 9.4 (SAS Institute, Inc., Cary, NC, USA), with the assistance of a professional statistician. The data were processed using descriptive statistics. The reliability of the instruments used (JSS and the three MBI subscales) was assessed using Cronbach's alpha coefficient, with coefficients in the 0–1 range and values equal to or greater than .70 indicating reliability.

The chi-square test was used to compare the qualitative variables of the MBI and the JSS. Modified adjusted Poisson regression models with robust variance (Zou, 2004) were constructed with the objective of verifying the relationship between a set of independent variables and the dependent variable regarding the occurrence of sleep disorder and the occurrence of high and moderate levels of EE, DE, and PA.

The results are presented using the means of the estimates obtained from the adjusted prevalence ratio with their respective 95% confidence intervals and *p* values, considering a significance level of 5% (*p* < .05).

## Results

A total of 577 nursing professionals agreed to participate in this study and responded to the questionnaire. However, five questionnaires were excluded because not all of the questions were answered. Therefore, 572 participants completed the questionnaire, of which 508 (88.8%) were women and 64 were men (11.2%), with a mean age of 36.4 years (*SD =* 8.8), ranging from 19 to 65 years. The region with the largest number of participants was the Southeast (71.7%), followed by the North (9.3%), the Central West (9.3%), the South (6.1%), and the Northeast (3.7%). With regard to professional training, the majority of the participants had completed specialization courses (52.1%).

With regard to work, 409 (71.5%) were nurses, 149 (26.1%) were nursing technicians, and 14 (2.4%) were nursing assistants. Most worked in tertiary care (68.9%), had a workload of 31 to 40 hours per week (45.8%), and were responsible for one job (69.2%).

Increased tensions and stress among shift team members were reported by 561 (98.1%) of the participants, and 462 (80.8%) reported an increase in the number of patients and the care to be performed during the pandemic. Sleep was self-rated as bad and very bad by 67.1%, followed by good (29.4%), and excellent (3.5%). The highest prevalence of bad and very bad sleep was reported among the nursing assistants (85.7%), followed by nursing technicians (69.1%) and nurses (65.5%). The responses showed that 25.9% of the professionals had started to use medication to sleep during the pandemic period.

The prevalence of sleep disorders assessed using the JSS was 26.4%. The sociodemographic and work variables and the changes reported during the pandemic according to the occurrence of sleep disorders measured through the JSS are presented in Table 1.

**Table 1.** Distribution of Sociodemographic and Work Variables, and Changes Reported During the Pandemic According to Sleep Disorders Measured Using the Jenkins Sleep Scale (*N* = 572)

Variable	Disorder According to the Jenkins Sleep Scale			
	No (*n =* 421)		Yes (*n =* 151)	
	*n*	%	*n*	%
Gender				
Female	368	72.4	140	27.6
Male	53	82.8	11	17.2
Professional category				
Nursing assistant	7	50.0	7	50.0
Nursing technician	108	72.5	41	27.5
Nurse	306	74.8	103	25.2
Jobs				
One	293	74.0	103	26.0
Two or more	128	72.7	48	27.3
Weekly workload (hours)				
20–30	49	71.0	20	29.0
31–40	198	75.6	64	24.4
41–50	84	73.0	31	27.0
> 50	90	71.4	36	28.6
Increase in the number of patients and care to be performed observed				
No	90	81.8	20	18.2
Yes	331	71.6	131	28.3
Increased tension and stress observed among shift team members				
No	8	72.7	3	27.3
Yes	413	73.6	148	26.4
Started using sleep medications				
No	346	81.6	78	18.4
Yes	75	50.7	73	49.3

The adjusted analysis carried out using a modified Poisson multiple regression with robust variance showed that the factors related to the JSS (Table 2) included the professional category and starting to use sleep medication.

**Table 2.** Multiple Poisson Regression for the Prevalence of Sleep Disorders Using the Jenkins Sleep Scale (*N* = 572)

Independent Variable	*PR* ª	95% CI	*p*
Gender			
Female (ref.: male)	1.61	[0.92, 2.81]	.092
Professional category			
Nurse (ref.: nursing assistant)	0.64	[0.43, 0.95]	.025
Nursing technician (ref.: nursing assistant)	0.66	[0.43, 1.03]	.066
Nursing technician (ref.: nurse)	1.04	[0.78, 1.39]	.794
Jobs			
Two or more (ref.: one)	1.00	[0.65, 1.53]	.986
Weekly workload (hours)			
> 50 (ref.: 20–30)	1.04	[0.58, 1.87]	.900
> 50 (ref.: 31–40)	1.18	[0.72, 1.95]	.511
> 50 (ref.: 41–50)	1.10	[0.66, 1.82]	.711
41–50 (ref.: 20–30)	0.94	[0.59, 1.52]	.812
41–50 (ref.: 31–40)	1.07	[0.75, 1.54]	.694
31–40 (ref.: 20–30)	0.88	[0.58, 1.33]	.541
Increase in the number of patients and care to be performed observed			
Yes (ref.: no)	1.44	[0.97, 2.15]	.071
Increased tension and stress observed among shift team members			
Yes (ref.: no)	0.70	[0.27, 1.81]	.464
Started using sleep medications			
Yes (ref.: no)	2.55	[1.96, 3.31]	< .001

***Note.*** Ref. = reference; PR = prevalence ratio; CI = confidence interval.

ª The prevalence of sleep disorders was estimated.

Burnout was identified in 17.3% of the sample. Mean MBI scores according to the EE, DE, and PA subscales were 28.0 (*SD* = 6.7), 10.1 (*SD* = 3.4), and 30.1 (*SD* = 4.4), respectively. Regarding the limits established for the burnout classification, for the EE subscale, the level was high in 57.3% of the participants, moderate in 37.6%, and low in 5.1%; for the DE subscale, the level was high in 23.8% of the participants, moderate in 60.8%, and low in 15.4%; and for the PA subscale, the level was high in 61.0% of the participants, moderate in 36.4%, and low in 2.6%.

As shown in Table 3, the male participants and participants with a weekly workload of ≥ 50 hours experienced a higher prevalence of high and moderate levels of EE and feelings of DE as well as lower levels of PA-related burnout.

**Table 3.** Distribution of Sociodemographic and Work Variables, and Changes Reported During the Pandemic According to the Level of Burnout Measured Using the Maslach Burnout Inventory Subscales (*N* = 572)

Variable	Emotional Exhaustion				Depersonalization				Personal Accomplishment			
	High/Medium		Low		High/Medium		Low		High/Medium		Low	
	*n*	%	*n*	%	*n*	%	*n*	%	*n*	%	*n*	%
Gender												
Female	481	94.7	27	5.3	426	83.9	82	16.1	495	97.4	13	2.6
Male	62	96.9	2	3.1	58	90.6	6	9.4	62	96.9	2	3.1
Professional category												
Nursing assistant	12	85.7	2	14.3	14	100.0	–	–	14	100.0	–	–
Nursing technician	135	90.6	14	9.4	126	84.6	23	15.4	144	96.6	5	3.4
Nurse	396	96.8	13	3.2	344	84.1	65	15.9	399	97.6	10	2.4
Jobs												
One	372	93.9	24	6.1	333	84.1	63	15.9	391	98.7	5	1.3
Two or more	171	97.2	5	2.8	151	85.8	25	14.2	166	94.3	10	5.7
Weekly workload (hours)												
20–30	61	88.4	8	11.6	58	84.1	11	15.9	67	97.1	2	2.9
31–40	247	94.3	15	5.7	211	80.5	51	19.5	258	98.5	4	1.5
41–50	111	96.5	4	3.48	100	87.0	15	13.0	112	97.4	3	2.6
> 50	124	98.4	2	1.2	115	91.3	11	8.7	120	95.2	6	4.8
Increase in the number of patients and care to be performed observed												
No	97	88.2	13	11.8	93	84.5	17	15.4	107	97.3	3	2.7
Yes	446	96.5	16	3.5	391	84.6	71	15.4	450	97.4	12	2.6
Increased tension and stress observed among shift team members												
No	7	63.6	4	36.4	10	90.9	1	9.1	10	90.9	1	9.1
Yes	536	95.5	25	4.5	474	84.5	87	15.5	547	97.5	14	2.5
Started using sleep medications												
No	397	93.6	27	6.4	350	82.5	74	17.4	409	96.5	15	3.5
Yes	146	98.6	2	1.3	134	90.5	14	9.5	148	100.0	–	–

As shown in Table 4, the prevalence of high and moderate EE was lower among nursing technicians than nurses (*PR* = 0.93), higher among those who observed an increase in the number of patients and the amount of care performed (*PR* = 1.07), and higher among those who started using sleep medication (*PR* = 1.05). The highest prevalence of high and moderate DE was observed in participants who had started using medication to sleep (*PR* = 1.08) and in those who worked ≥ 50 hours per week compared to those who worked 20 to 30 hours per week (*PR* = 1.15) and 31 to 40 hours per week (*PR* = 1.19). Furthermore, a high level of PA-related burnout was observed among those who had started using medication (*PR* = 1.03).

**Table 4.** Multiple Poisson Regression for the Prevalence of High and Moderate Burnout Levels Using the Subscales of the Maslach Burnout Inventory (*N* = 572)

Independent Variable	Emotional Exhaustion			Depersonalization			Personal Accomplishment		
	*PR* ^a^	95% CI	*p*	*PR* ^b^	95% CI	*p*	*PR* ^c^	95% CI	*p*
Gender									
Female (ref.: male)	0.98	[0.93, 1.03]	.493	0.95	[0.87, 1.04]	.229	0.99	[0.94, 1.05]	.842
Professional category									
Nurse (ref.: nursing assistant)	1.05	[0.91, 1.20]	.497	0.86	[0.80, 0.92]	<.001	0.97	[0.93, 1.02]	.206
Nursing technician (ref.: nursing assistant)	0.97	[0.85, 1.12]	.719	0.87	[0.79, 0.95]	<.001	0.96	[0.91, 1.01]	.138
Nursing technician (ref.: nurse)	0.93	[0.88, 0.98]	.006	1.01	[0.93, 1.09]	.827	0.99	[0.96, 1.02]	.532
Jobs									
Two or more (ref.: one)	1.00	[0.94, 1.06]	.959	0.92	[0.83, 1.02]	.133	0.95	[0.90, 1.00]	.058
Weekly workload (hours)									
> 50 (ref.: 20–30)	1.10	[0.99, 1.22]	.071	1.15	[1.01, 1.31]	.041	1.02	[0.95, 1.09]	.667
> 50 (ref.: 31–40)	1.04	[0.97, 1.10]	.266	1.19	[1.06, 1.34]	.002	1.00	[0.94, 1.06]	.966
> 50 (ref.: 41–50)	1.01	[0.95, 1.07]	.778	1.10	[0.99, 1.21]	.078	1.01	[0.95, 1.06]	.838
41–50 (ref.: 20–30)	1.09	[1.00, 1.19]	.056	1.05	[0.93, 1.19]	.458	1.01	[0.96, 1.06]	.707
41–50 (ref.: 31–40)	1.03	[0.98, 1.07]	.238	1.09	[0.99, 1.20]	.084	1.00	[0.96, 1.03]	.793
31–40 (ref.: 20–30)	1.06	[0.97, 1.15]	.172	0.96	[0.86, 1.08]	.520	1.01	[0.97, 1.06]	.504
Increase in the number of patients and care to be performed observed									
Yes (ref.: no)	1.07	[1.01, 1.15]	.033	1.01	[0.92, 1.11]	.809	1.00	[0.97, 1.03]	.954
Increased tension and stress observed among shift team members									
Yes (ref.: no)	1.46	[0.95, 2.24]	.085	0.96	[0.78, 1.17]	.681	1.09	[0.90, 1.32]	.381
Started using medications to sleep									
Yes (ref.: no)	1.05	[1.02, 1.08]	.003	1.08	[1.01, 1.16]	.021	1.03	[1.02, 1.05]	< .001

***Note.*** Ref. = reference; PR = prevalence ratio; CI = confidence interval.

^a^ The prevalence of high and moderate levels of emotional exhaustion was estimated. ^b^ The prevalence of high and moderate levels of depersonalization was estimated; the prevalence of high and moderate levels of personal accomplishment was estimated.

Based on the JSS, in terms of sleep disorders, a difference in level of burnout was identified among the EE, DE and PA subscales of the MBI. Among the participants with sleep disorders, all presented high and moderate levels of EE and high levels of PA-related burnout (Table 5).

**Table 5.** Distribution of the Subscales of the Maslach Burnout Inventory According to the Presence of Sleep Disorders Measured Using the Jenkins Sleep Scale (*N* = 572)

Variable (Maslach Burnout Inventory Subscale)	Sleep Disorder According to the Jenkins Sleep Scale				*p* ^a^
	No (*n* = 421)		Yes (*n* = 151)		
	*n*	%	*n*	%	
Emotional exhaustion					< .001
High and moderate level	392	72.2	151	27.8	
Low level	29	100.0	0	0	
Depersonalization					< .001
High and moderate levels	345	71.3	139	28.7	
Low level	76	86.4	12	13.6	
Personal accomplishment					.002
High and moderate levels	406	72.9	151	27.1	
Low level	15	100.0	0	0	

^a^*p* value obtained using the chi-square test.

## Discussion

The current burden carried by nursing professionals, including increased tension and stress among team members and the increased amounts of care burden, may trigger increased fatigue. Working conditions impact the health of workers in the healthcare profession. Horesh and Brown (2020) highlighted that researchers and professionals specializing in mental health should respond to this pressing need by developing appropriate methods and tools to reduce the rates of post-traumatic stress, anxiety, and depression among healthcare professionals working in COVID-19 pandemic conditions to help them minimize the bad feelings that often arise during the patient care process. Furthermore, poor quality of sleep and stress arising from direct contact between healthcare workers and COVID-19 increase their risk of infection from this disease (Wang et al., 2020).

In this study, 26.4% of the participants presented scores indicative of sleep disorders, according to the JSS. Studies have shown that sleep disorders are frequent and that their prevalence is higher in healthcare providers than in the general population, and that, as a result of the pandemic, sleep complaints have intensified (Aydin Sayilan et al., 2021; Beck et al., 2021; Qiu et al., 2020; Salari et al., 2020). In Spain, a study that included 100 healthcare workers and 70 non-healthcare workers found that insomnia developed or worsened in 57.0% of the healthcare workers compared to 34.2% in the group of non-healthcare workers (Herrero San Martin et al., 2020). In addition to sleep disorders, poor sleep quality is described as common in nursing care providers (Khatony et al., 2020; Zeng et al., 2020). An important finding of this study is that most participants reported poor sleep quality, with a higher prevalence among nursing assistants. This situation may be due to the characteristics of the activities performed by these professionals, together with the precarious job market (Silva et al., 2020). Continuous and prolonged work very close to patients, multiple and exhausting work shifts, low levels of autonomy, dissatisfaction with work responsibilities, and lack of preparation to face emotional issues are some particularities inherent to the work of nursing assistants and nursing technicians that make them more susceptible to developing sleep problems. However, it is necessary to be cautious with this finding because of the limited number of participants in these professional categories (particularly nursing assistants) included in this study. Most studies have been conducted on nurses, who typically present a variety of complaints. However, the results of this study suggest that nursing assistants and technicians may present even more problems than nurses, highlighting the relevance of studying these subcategories further.

In this study, sleep was self-rated as bad or very bad by 54.9% of the participants. Other studies have reported a similar finding namely. For example, poor quality sleep has been identified in 77.4% of nurses (Khatony et al., 2020), and 64.0% of health professionals presented poor quality sleep compared to 31.0% in a group of non-workers (Herrero San Martin et al., 2020). Zhou et al. (2020), analyzing a sample of 1,931 frontline health professionals during the COVID-19 pandemic, found a lower prevalence of poor sleep quality (18.4%) and a positive association between being a nurse (*OR* = 3.132, 95% CI [1.727, 5.681], *p* < .001) and poor sleep quality. In nurses with insufficient sleep, short sleep duration, and insomnia, the chance of assessing their own health as poor (*OR* = 4.49, 95% CI [3.25, 6.22]) was greater than of those with adequate sleep duration and no complaints regarding sleep quality (Silva-Costa et al., 2015). Poor sleep quality and sleep disorders are important health indicators that may influence quality of care, and increase the risk of errors (Weaver et al., 2016), as well as negatively impact the team. Furthermore, occupational demands are frequent causes of insufficient sleep and may elevate the risk of accidents at work (Mukherjee et al., 2015).

Sleep is a modifiable factor, with good sleep quality having been proposed as a protective factor for the health and well-being of nursing professionals as well as a factor promoting quality of care, as observed in hospitals in the United States (Lee et al., 2021).

Nearly all of the participants reported perceiving an increase in workload and tension and stress among team members during the COVID-19 pandemic. These changes may contribute to sleep disorders and professional burnout. In a study conducted in China on 1,257 healthcare workers during the COVID-19 pandemic, 70.0% reported suffering, 50.0% reported depression, 44.6% reported symptoms of anxiety, and 34.0% reported insomnia (Lai et al., 2020). In situations such as the pandemic, which is considered a public health problem, the factors listed above should receive attention, as they may be exacerbated (Sher, 2020) and are generally associated with impaired sleep quality.

Another factor to consider is that approximately 26.0% of the sample started using medication to sleep, which suggests negative changes in sleep quality, for which these professionals seek solutions that may have an unwanted impact on their health (Beck et al., 2021). Pandemic-related changes in sleep have increased the use of medications to improve sleep quality in healthy people (Salehinejad et al., 2020). The stress of living with the insecurity of a new disease has imposed a new routine on the teams, who have had their performance affected by their extra work, generating harmful personal physical and emotional consequences.

In this study, burnout was identified in 17.3% of the participants, and, when analyzing the mean scores for the MBI subscales, it was observed that these indicated a high level of burnout in EE (28.0), a moderate level in DE (10.1), and a high level in PA (30.1). Exhaustion, negative attitudes, dissatisfaction with their own performance, physical symptoms, and insomnia are commonly reported by workers with burnout syndrome (Salvagioni et al., 2017). Khasne et al. (2020) found a high level of exhaustion related to the pandemic in healthcare workers, with less personal and professional exhaustion for the support team, which included nursing assistants. The performance of a professional activity should be a source of satisfaction and should reaffirm the importance of the role of this individual in society. However, work overload and different levels of stress can negatively impact the health of nurses leading to burnout (Nogueira et al., 2018).

The participants in this study were working in an unfavorable environment that included direct contact with patients with COVID-19, uncertainty about the possibility of contagion, long hours of work, and the stress of the entire team. Therefore, it is possible that exposure to this environment increased their symptoms of burnout.

It was found that men and those with the greatest weekly workload reported higher levels of EE and DE and lower levels of PA-related burnout. Evidence in the literature suggests that gender influences level of burnout. Typically, women experience more EE, while men are more prone to DE (Cañadas-De et al., 2015), although there is no consensus in the literature. Relatively high levels of burnout in men have been previously reported (Cañadas-De et al., 2018). In this study, 43.8% of the men reported a weekly workload of ≥ 50 hours (data not shown), which may contribute to this finding, as work overload has been identified as a trigger for burnout (Maslach et al., 2001) and may have a deleterious effect on health. It is necessary to consider that it is increasingly common for men and women to share care of the home and children, in addition to professional work, which may constitute an additional factor influencing emotional tension. A recent systematic review (Chemali et al., 2019) revealed that the factors commonly associated with high levels of burnout include heavy work, a disorganized environment, excessive working hours, and difficulty in reconciling professional and private aspects of life. Working conditions during the pandemic, the additional hours worked, and the additional workload due to the shortage of professionals in Brazil may have contributed to the results in this study.

In this study, a significant association was found between level of burnout (EE and DE subscales) and professional category. Other authors have also reported a relationship between EE and professional category (Baldonedo-Mosteiro et al., 2019). The significant relationship between sleep disorder and burnout scores should be viewed with caution, as studies have shown that impaired sleep compromises professional performance, quality of life, and health and impacts on mental health (Aydin Sayilan et al., 2021; Kang et al., 2020; Qiu et al., 2020; Rajkumar, 2020; Wang et al., 2020). Accordingly, health team managers should focus adequate attention on these variables, as they are responsible for implementing measures to evaluate and improve the working conditions for the nursing team to reduce nurses' professional burnout and optimize their sleep quality. Programs to prevent sleep disorders and professional exhaustion may be planned. The efficacy of these interventions should be assessed in future studies.

### Methodological Issues and Limitations

This study was subject to several limitations. The first was the cross-sectional design used, which does not allow for the evaluation of variables over time or of causal relationships between them. The second was the fact that the sleep disorders and physical and emotional exhaustion were assessed using self-report instruments, which may be less accurate in identifying symptoms. Furthermore, the participants were not asked about the presence of a primary sleep disorder. The third was that the minimum sample size was not achieved, which may have compromised the statistical significance of the findings.

Applying questionnaires that have been widely used in the literature to help reduce the subjectivity evaluations and allow direct comparisons with the findings of other authors may help mitigate these limitations. The instruments used in this study were shown to provide satisfactory internal consistency, with Cronbach's alphas greater than .70. The DE subscale had a reliability of .63, indicating caution in using its results.

### Conclusions and Implications for Practice

Sleep disorders and burnout were found in a significant proportion of the nursing professionals who participated in this study. The factors associated with sleep disorders according to the JSS included the professional category, with a lower prevalence of disorders among nurses than nursing assistants and among those who began using medications to sleep during the pandemic.

Higher levels of EE-related burnout were significantly associated with the nursing technician professional category, in those who experienced increases in the number of patients cared for and the amount of necessary care, and in those who had begun using sleep medication. With regard to DE-related burnout, a significantly higher level was observed in nursing assistants, in those with a weekly workload of 50 hours or more, and in those who had begun using sleep medication. Finally, a significantly higher level of PA-related burnout was found in those who had begun using sleep medication.

Nursing professionals, especially nursing technicians and assistants, should be evaluated periodically for sleep disorders and burnout, with additional care provided during abnormal periods such as the current pandemic. Furthermore, starting to use medication to promote sleep may have negative effects on health, quality of life, and safety at work, which should be of concern to health administrators. Interventions to enhance the work environment and sleep quality should be implemented to improve the physical and mental health of nursing professionals and sustain quality of care. It is important to disseminate information and guidance about burnout and sleep problems and their consequences for nursing workers, propose preventative actions, monitor for early recognition of signs and symptoms, provide guidance on changing lifestyles, and offer referrals when treatment is necessary.
